# Differential gene expression orchestrated by transcription factors in osteoporosis: bioinformatics analysis of associated polymorphism elaborating functional relationships

**DOI:** 10.18632/aging.204136

**Published:** 2022-06-21

**Authors:** Chih-Chien Wang, Jen-Jie Weng, Hsiang-Cheng Chen, Meng-Chang Lee, Pi-Shao Ko, Sui-Lung Su

**Affiliations:** 1 Department of Orthopedics, Tri-Service General Hospital and National Defense Medical Center, Taipei, Taiwan, R.O.C.; 2 School of Public Health, National Defense Medical Center, Taipei, Taiwan, R.O.C.; 3 Division of Rheumatology, Immunology and Allergy, Department of Internal Medicine, Tri-Service General Hospital, National Defense Medical Center, Taipei, Taiwan, R.O.C.; 4 Graduate Institute of Life Sciences, National Defense Medical Center, Taipei, Taiwan, R.O.C.

**Keywords:** osteoporosis, transcription factor binding site, bioinformatics

## Abstract

Background: Identification of candidate SNPs from transcription factors (TFs) is a novel concept, while systematic large-scale studies on these SNPs are scarce.

Purpose: This study aimed to identify the SNPs of six TF binding sites (TFBSs) and examine the association between candidate SNPs and osteoporosis.

Methods: We used the Taiwan BioBank database; University of California, Santa Cruz, reference genome; and a chromatin immunoprecipitation sequencing database to detect 14 SNPs at the potential binding sites of six TFs. Moreover, we performed a case–control study and genotyped 109 patients with osteoporosis (T-score ≤ −2.5 evaluated by dual-energy X-ray absorptiometry) and 262 healthy individuals (T-score ≥ −1) at Tri-Service General Hospital from 2015 to 2019. Furthermore, we used the expression quantitative trait loci (eQTL) from the Genotype-Tissue Expression database to identify downstream gene expression as a criterion for the function of candidate SNPs.

Results: Bioinformatic analysis identified 14 SNPs of TFBSs influencing osteoporosis. Of these SNPs, the rs130347 CC + TC genotype had 0.57 times higher risk than the TT genotype (OR = 0.57, p = 0.031). Validation of eQTL analysis revealed that rs130347 T allele influences mRNA expression of downstream *A4GALT* in whole blood (p = 0.0041) and skeletal tissues (p = 0.011).

Conclusions: We successfully identified the unique osteoporosis locus rs130347 in the Taiwanese and functionally validated this finding. In the future, this strategy can be expanded to other diseases to identify susceptible loci and achieve personalized precision medicine.

## INTRODUCTION

Osteoporosis is a disease of the skeletal system, characterized by decreases in bone mass and density and structural deterioration, leading to bone fragility and increased fracture risk [1]. Globally, there are currently at least 200 million patients with osteoporosis. The prevalence of osteoporosis increases with aging in a population, particularly in female [2]. Data from the National Health Insurance Research Database in Taiwan revealed that the overall prevalence of osteoporosis in people aged ≥50 years increased from 17.4% to 25% in the period of 2001-2011 [3]. The prevalence of osteoporosis in males increased from 6.9% to 13.3%—and that of osteoporosis in females increased from 28.1% to 36.2% [3].

Bone mineral density (BMD) is a complex characteristic influenced by individual, environmental, and genetic factors. Aging, gender differences, vitamin D and calcium intake, smoking, and alcohol consumption influence bone mass and BMD [4–7]. Approximately 50%–80% of the decrease in BMD can be attributed to genetics and result in osteoporosis [8–11].

Genome-wide association study (GWAS) has become a widely used approach in genome research. This method is based on the use of linkage disequilibrium in chromosomes to map disease-causing alleles [12]. However, for several gene loci, despite repeated analyses by GWAS, their association with complex diseases has remained unclear [13]. An example has shown GWAS in osteoporosis-related study could only explain approximately 20% of the variation in BMD due to missing heritability and stringent statistical test value (p < 5 × 10^−8^) [14, 15]. The pathophysiological causes of osteoporosis are complex, with orchestration of various transcription factor (TF) and biological pathways, forming a complex regulatory network [16, 17]. A previous study used the GSE35958 database to analyze differentially expressed genes (DEGs) in patients with osteoporosis and a control group and identified the following osteoporosis-related TFs: E2F TF 4 (E2F4), early growth response 1 (EGR1), *JUN* proto-oncogene (JUN), trans-acting TF 1 (Sp1), TF 7-like 2 (TCF7L2), tumor protein p53 (TP53), and catenin (cadherin-associated protein) beta 1 (CTNNB1) [18, 19]. To our knowledge, polymorphisms in TF binding sites (TFBSs) have been explored their association with disease [20]. As a result, we aligned Taiwan BioBank polymorphisms database to binding sites of 7 chosen TFs by conserved motifs to seek potential disease-related single-nucleotide polymorphisms (SNPs) in Taiwanese. We performed a case–control study to investigate the association between the candidate SNPs and osteoporosis. Finally, we used the Genotype-Tissue Expression (GTEx) database to validate the functionality of SNPs.

## MATERIALS AND METHODS

### Study participants

This case–control study was conducted between March 2015 and October 2019. In this study, 371 postmenopausal women (109 patients with osteoporosis and 262 healthy individuals) were enrolled from Tri-Service General Hospital (TSGH). None of the participants had any history of medication for treating osteoporosis. Data on the demographic and clinical characteristics of all participants were obtained from questionnaires and medical records.

### Bone mineral density measurements

BMD (g/cm^2^), an indicator of osteoporosis, calculated by dividing the bone mineral content (g) by the bone area (cm^2^) [21], of all participants was measured using dual-energy X-ray absorptiometry (DXA) (GE Medical Systems Lunar, Madison, WI, USA) [22] at the lumbar spine 1–4, and the diagnosis of osteoporosis was based on the World Health Organization (WHO) standards. By osteoporosis is meant BMD measurements at or below the −2.5 standard deviation (SD) from the optimal peak bone density (T-score) of a healthy young adult of the same sex; by contrast, BMD measurement at or above −1 SD from T-score of a healthy young adult of the same sex was considered to reflect bone mass normal [23].

### Bioinformatic analysis in gene screening

### Screening procedures for genetic variation in Taiwanese

We referred to a previous study conducted by Xie et al. [19], which used microarray data to analyze DEGs and obtained seven osteoporosis-related TFs: E2F4, EGR1, JUN, Sp1, TCF7L2, TP53, and CTNNB1. Subsequently, we used the next-generation sequencing (NGS) data of 1,517 people; the data were available from the Taiwan BioBank database and included 74,861,556 genetic variants. We excluded the structural variants (insertions/deletions) because it was not available to use a multifunctional mass spectrometer (mass array) for genotyping. Then, we excluded the SNPs with a call rate of <90%. Finally, we used the chosen SNPs for further alignment.

### Identifying the genetic variants that may influence TF binding

First, we used a human reference genome sequence downloaded from the University of California, Santa Cruz (UCSC; GRCh37/hg19). We analyzed genetic variants that may influence TF binding by using bioinformatic sequence alignment techniques and identified the variants located in the TFBS.

Alignment of the binding site of TF E2F4—5′-TTTSSCGC-3′ (S = C or G)—in all 52,392,270 SNPs derived from the Taiwan BioBank database revealed 12,124 SNPs that may influence the binding affinity: EGR1—5′-GCGGGGGCGG-3′— 689 SNPs; JUN—5′-TGASTCA-3′— 81,754 SNPs; Sp1—5′-GGGCGG-3′— 97,654 SNPs; TCF7L2—5′-CTTTGA-3′— 169,619 SNPs; TP53—5′-RRRCWWGYYY-3′ (R = A or G;W = A or T; Y = C or T)— 169,319 SNPs; and CTNNB1, 5′—TGAYTCA-3′— 84,951 SNPs. We selected SNPs with a minor allele frequency (MAF) <5% as the SNP fragments that influence the TFBSs. The code used for bioinformatics sequence alignment in R is shown in Supplementary File 1.

### Confirming the TF binding affinity of the genetic variants through ChIP-Seq

**Figure 1:**
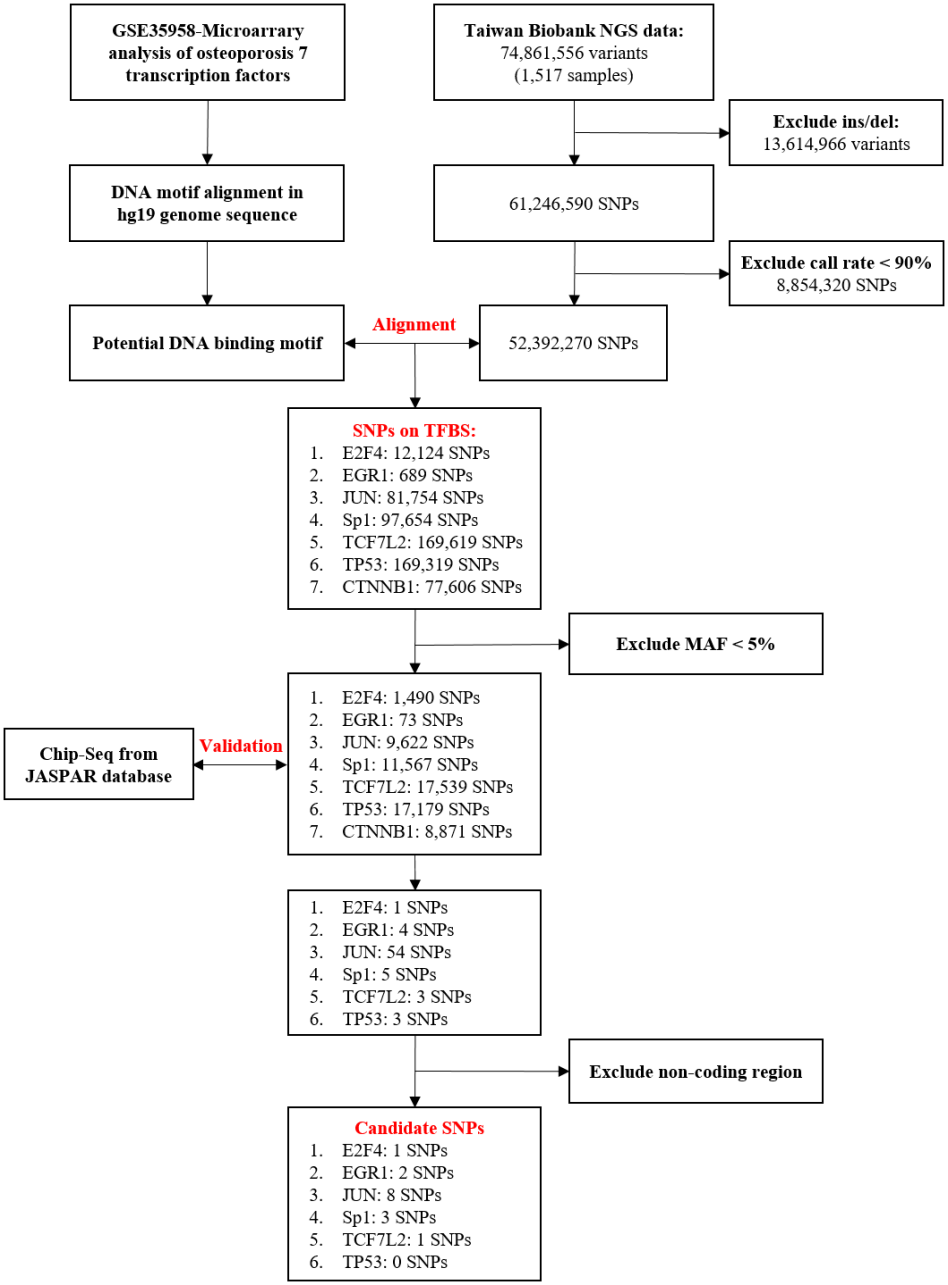
**Flowchart of the stepwise approach to screen for candidate transcription factors (TFs) and binding site SNPs.** Upstream predictors of seven TFs, E2F4, EGR1, JUN, Sp1, TCF7L2, TP53, and CTNNB1, in osteoporosis [19]. Identification of genetic variants that may influence TFBS through bioinformatic sequence alignment. First, we used the data of a total of 74,861,556 variants (1,517 samples) obtained from the Taiwan BioBank database to screen for Taiwanese population-specific genetic variation. Then, through genetic alignment of GRCh37/hg19 obtained from the National Center for Biotechnology Information database, we found SNPs that may influence the binding affinity. SNPs with an MAF of <5% were excluded from the samples. Chromatin immunoprecipitation sequencing (ChIP-Seq) data obtained from the JASPAR database were used to confirm whether these genetic variants had a combination of the sites. No ChIP-Seq data were available for CTNNB1 validation, and this gene was thus excluded. Finally, we excluded results of the noncoding regions. The variation of 14 SNPs may influence transcription factor binding activity. DEG, differentially expressed gene; NGS, next-generation sequencing; SNP, single-nucleotide polymorphism; Ins/del, insertion/deletion; TFBS, TF binding site; MAF, minor allele frequency.

ChIP-Seq data from the JASPAR database were used to confirm whether the genetic variants had a combination of the following sites [24]: E2F4 (Matrix ID: MA0470.1) [25], EGR1 (Matrix ID: MA0162.2) [26], JUN (Matrix ID: MA0488.1) [27], Sp1 (Matrix ID: MA0079.3) [28], TCF7L2 (Matrix ID: MA0523.1) [29], and TP53 (Matrix ID: MA0106.2) [30]. As ChIP-Seq data were not available for CTNNB1, further examination of this TF did not perform in this study. Finally, a total of 14 SNPs were successfully selected: rs55785541, rs2295624, rs79436692, rs1243673, rs6108246, rs6688233, rs130347, rs6509294, rs3758354, rs117405516, rs3813600, rs3803353, rs77796751, and rs28481460 (Figure 1).

### Genomic DNA extraction and SNP genotyping

Genomic DNA was isolated from the peripheral blood samples using the standard procedures for proteinase K (Invitrogen, Carlsbad, CA, USA) digestion and the phenol/chloroform method [31]. 14 SNPs, mentioned above, in the TFBSs were genotyped by iPLEX Gold SNP genotyping [32], a genotyping example, rs55785541, shown in Supplementary Figure 1. We used inter-replication validation to assess the quality of the genotyping experiment, which was performed with 19 replicate samples (approximately 5%), and the concordance rate was 100%.

### Ethics

This study was reviewed and approved by the institutional ethics committee of TSGH (B202105044). After a detailed explanation of the study objectives, written informed consent was obtained from all participants. All clinical and biological samples were collected and DNA was genotyped after obtaining patient consent.

### Statistical analysis

Continuous variables were reported as the mean ± SD and were tested using t-tests and ANOVA. Genotype and allelic frequencies were compared between patients with osteoporosis and healthy individuals using chi-squared or Fisher’s exact test. Logistic regression analysis was performed to estimate odds ratios (ORs) and 95% confidence intervals (CIs) [13] with adjustment for age and gender. The analysis was performed using allele type, genotype, dominant, and recessive models. Statistical analyses were performed using SPSS 22.0 (SPSS Inc., Chicago, IL, USA) and R 3.5.1 (R Project for Statistical Computing, Vienna, Austria). A p-value of <0.05 was considered statistically significant.

## RESULTS

### Candidate TFs and polymorphic TFBSs

According to Xie et al. [19], upstream TFs, E2F4, EGR1, JUN, Sp1, TCF7L2, TP53 and CTNNB1, were identified from DEGs associated with osteoporosis. We then selected NGS data on 1,517 samples from the Taiwan BioBank and excluded 13,614,966 SNPs with structural mutations (insertions/deletions) and 8,854,320 SNPs with a sequencing quality control call rate of <90%. As a result, 52,392,270 SNPs were included in this study.

The UCSC human genomic sequence hg19 and homologous or motif sequences were used for sequence alignment with the SNPs screened from the Taiwan BioBank. The TFs E2F4, EGR1, JUN, Sp1, TCF7L2, TP53, and CTNNB1 had 12,124, 689, 81,754, 97,654, 169,619, 169,319, and 77,606 SNPs, respectively, on the binding sites. After excluding the sites with an MAF of <5%, 1,490, 73, 9,622, 11,567, 17,539, 17,179, and 8,871 binding sites remained, respectively, for E2F4, EGR1, JUN, Sp1, TCF7L2, TP53, and CTNNB1. This was followed by repeated validation using ChIP-Seq data and the exclusion of noncoding regions. Finally, for E2F4, EGR1, JUN, Sp1, TCF7L2, and TP53, 1, 2, 8, 3, 1, and 0 SNPs, respectively, were successfully included in this study. For CTNNB1, as ChIP-Seq data were not available for validation, no SNP was included in this study. In summary, 14 sites in the abovementioned results were included in this study. The detailed candidate results are shown in Table 1.

**Table 1:** Summary of three candidate SNPs obtained from bioinformatics analyses.

**SNPs**	**Chromosome:Position**	**TF**	**MAF**	**Gene**	**DNA sequence near the SNP**
rs55785541	15:100890478	E2F4	0.20 (G/A)	*SPATA41*	CTTTCCC**[G/A]**CGTCAGC
rs2295624	1:229644157	EGR1	0.17 (G/T)	*ABCB10*	GCGGGGGCG**[G/T]**GATCGTGAC
rs79436692	15:41576159	EGR1& SP1	0.16 (G/A)	*OIP5-AS1*	CAGCGGCGG**[G/A]**GGCGGGGCT
rs12463673	2:43412531	JUN	0.23 (C/T)	*ZFP36L2*	AATGAG**[C/T]**CAGAGA
rs6108246	20:9032379	JUN	0.14 (T/G)	*PLCB4*	GTTTAT**[T/G]**ACTCAT
rs6688233	1:9335745	JUN	0.21(C/T)	*SPSB1*	CTGACT**[C/T]**ATAGCT
rs130347	22:43076809	JUN	0.27(C/T)	*A4GALT*	CTGCAC**[C/T]**GAGTCA
rs6509294	19:47323384	JUN	0.10(G/A)	*SNAR-E*	TGAGTC**[G/A]**TGGTGA
rs3758354	9:75764565	JUN	0.17 (A/C)	*ANXA1*	CGATGA**[A/C]**TCATCA
rs117405516	17:42983641	JUN	0.09(G/A)	*GFAP*	GGATGA**[G/A]**TCACTT
rs3813600	1:85786166	JUN	0.23(G/A)	*LOC646626*	TACGGT**[G/A]**AGTCAG
rs3803353	15:40857240	SP1	0.10(G/A)	*C15orf57*	GGGAG**[G/A]**GGCGG
rs77796751	5:137878943	SP1	0.05(G/A)	*ETF1*	GCCAG**[G/A]**GGCGG
rs28481460	15:89610555	TCF7L2	0.33(A/C)	*ABHD2*	CTTTG**[A/C]**AGCAT

TF, transcription factor; MAF, minor allele frequency (major/minor).

### Basic demographic analysis

Basic demographics are shown in Table 2. There were 262 healthy individuals in the control group and 109 patients in the osteoporosis group. The body mass index (BMI) of the osteoporosis group was lower than that of the control group (p < 0.001). The waist circumference of the osteoporosis group was lower than that of the control group (p < 0.001). Higher proportions of participants in the osteoporosis group took calcium tablets (p = 0.002) and suffered from knee osteoarthritis (p = 0.01).

**Table 2:** Basic demographic variables.

**Variable**	**Control group (N = 262)**	**Osteoporosis group (N = 109)**	**p-value**
Age (mean ± SD)	71.88 ± 6.48	72.03 ± 6.62	0.538
BMI (mean ± SD)	25.03 ± 3.74	22.19 ± 3.17	<0.001*
Waist circumference (mean ± SD)	81.85 ± 10.73	76.27 ± 8.85	<0.001*
Alcohol consumption, n (%)			0.461
No	254 (98.8)	105 (98.1)	
Yes	3 (1.2)	2 (1.9)	
Smoking status, n (%)			0.793
No	243 (97.6)	102 (99.0)	
Yes	6 (2.4)	1 (1.0)	
Periodic use of calcium tablets, n (%)			0.002*
No	181 (70.4)	58 (54.2)	
Yes	76 (29.6)	49 (45.8)	
Medical history, n (%)			
Hypertension	75 (28.6)	22 (20.2)	0.093
Diabetes	38 (14.5)	11 (10.1)	0.521
Knee osteoarthritis, n (%)	68 (26.0)	14 (12.8)	0.010*

Healthy individuals (control group): T-score ≥ −1; osteoporosis: T-score ≤ −2.5;

*:p-value < 0.05; BMI, body mass index.

### Association between binding site gene polymorphisms and osteoporosis susceptibility

In total, 14 SNPs were included in this study. All loci conformed to Hardy–Weinberg equilibrium (p > 0.05), with the exception of rs3813600 (p = 0.002). Genotyping results were obtained for 14 SNPs. Our results based on the genotype model showed that rs130347 SNP had a significant association with osteoporosis (p = 0.022; Table 3).

**Table 3:** Genotype distribution of TFBS SNPs in patients with osteoporosis and healthy individuals.

**SNPs**	**Controls (N=262), n (%)**	**Osteoporosis (N=109), n (%)**	**Crude-OR (95% CI)**	**p-value**	**Adj-OR (95% CI)^a^**	**p-value**
rs55785541				0.569		0.328
GG	154 (59.5)	58 (54.2)	1.00		1.00	
GA	95 (36.7)	43 (40.2)	1.20 (0.75–1.92)	0.443	1.29 (0.76–2.19)	0.339
AA	10 (3.9)	6 (5.6)	1.59 (0.55–4.58)	0.388	2.32 (0.66–8.21)	0.191
rs117405516				0.554		0.812
GG	220 (84.6)	94 (86.2)	1.00		1.00	
GA	38 (14.6)	13 (11.9)	0.80 (0.41–1.57)	0.518	0.91 (0.43–1.89)	0.791
AA	2 (0.8)	2 (1.8)	2.34 (0.32–16.86)	0.399	1.97 (0.20–19.88)	0.564
rs28481460				0.075		0.077
AA	180 (70.0)	32 (31.7)	1.00		1.00	
AC	69 (26.8)	57 (56.4)	1.80 (1.08–2.98)	0.023*	1.93 (1.09–3.42)	0.024*
CC	8 (3.1)	12 (11.9)	1.54 (0.70–3.38)	0.280	1.64 (0.68–3.92)	0.268
rs12463673				0.493		0.541
CC	131 (51.4)	61 (58.1)	1.00		1.00	
CT	105 (41.2)	38 (36.2)	0.78 (0.48–1.26)	0.303	0.74 (0.43–1.27)	0.27
TT	19(7.5)	6 (5.7)	0.68 (0.26–1.78)	0.431	0.93 (0.32–2.73)	0.901
rs130347				0.047*		0.022*
CC	108 (41.9)	53 (49.5)	1.00		1.00	
CT	126 (48.8)	38 (35.5)	0.61 (0.38–1.00)	0.051	0.48 (0.27–0.83)	0.009*
TT	24 (9.3)	16 (15.0)	1.36 (0.67–2.77)	0.400	1.05 (0.46–2.38)	0.905
rs6108246				0.991		0.964
GG	180 (71.7)	74 (71.8)	1.00		1.00	
GT	63 (25.1)	26 (25.2)	1.00 (0.59–1.71)	0.989	0.93(0.51–1.67)	0.803
TT	8 (3.2)	3(2.9)	0.91 (0.24–3.53)	0.894	1.06 (0.23–4.85)	0.939
rs6688233				0.420		0.397
CC	155 (59.6)	66(61.7)	1.00		1.00	
CT	97 (37.3)	35(32.7)	0.85 (0.52–1.37)	0.501	0.83 (0.48–1.44)	0.512
TT	8 (3.1)	6 (5.6)	1.76 (0.59–5.28)	0.312	1.92 (0.58–6.32)	0.285
rs6509294				0.656		0.479
GG	205 (80.7)	81 (76.4)	1.00		1.00	
GA	47 (18.5)	24 (22.6)	1.29 (0.74–2.25)	0.365	1.37 (0.73–2.58)	0.326
AA	2 (0.8)	1 (0.9)	1.27 (0.11–14.15)	0.848	2.75 (0.21–36.63)	0.443
rs3758354				0.338		0.495
AA	176 (68.0)	80 (74.8)	1.00		1.00	
AC	76 (29.3)	26 (24.3)	0.75 (0.45–1.26)	0.282	0.80 (0.45–1.44)	0.464
CC	7 (2.7)	1 (0.9)	0.31 (0.04–2.60)	0.283	0.34 (0.04–2.92)	0.324
rs3813600				0.656		0.623
GG	147 (56.8)	63 (58.3)	1.00		1.00	
GA	96 (37.1)	36 (33.3)	0.88 (0.54–1.42)	0.588	0.77 (0.45–1.32)	0.333
AA	16 (6.2)	9 (8.3)	1.31 (0.55–3.13)	0.539	0.95 (0.36–2.48)	0.920
rs22956524				0.631		0.582
GG	180 (70.0)	79 (74.5)	1.00		1.00	
GT	69 (26.8)	25 (23.6)	0.83 (0.49–1.40)	0.477	0.75 (0.42–1.36)	0.343
TT	8 (3.1)	2 (1.9)	0.57 (0.12–2.74)	0.483	0.65 (0.12–3.51)	0.615

*:p-value < 0.05; a, after the adjustment for age and body mass index; OR, odds ratio; CI, confidence interval.

In Table 4, we present the logistic regression analysis data comparing the genotype and allele frequencies of patients with osteoporosis and healthy individuals. For rs130347, a significant difference was found in the dominant model (CC vs. CT + TT) in all participants after adjustment for age and BMI (OR = 0.57, 95% CI = 0.34–0.95; p = 0.031). For rs28481460, a significant difference was found in the dominant model (AA + AC vs. CC) in all participants after adjustment for age and BMI (OR = 1.87, 95% CI = 1.08–3.24; p = 0.026).

**Table 4:** Association of rs130347 and rs28481460 with osteoporosis.

**Independent variable**	**Crude-OR (95% CI)**	**p-value**	**Adj-OR (95% CI)^a^**	**p-value**
rs130347				
Allele model		0.792		0.305
C	1.00		1.00	
T	0.96 (0.68–1.34)		0.82 (0.55–1.20)	
Dominant model		0.180		0.031*
CC	1.00		1.00	
CT + TT	0.73 (0.47–1.15)		0.57 (0.34–0.95)	
Recessive model		0.119		0.325
CC + CT	1.00		1.00	
TT	1.71 (0.87–3.37)		1.48 (0.68–3.22)	
rs28481460				
Allele model		0.078		0.03*
A	1.00		1.00	
C	1.36 (0.97–1.90)		1.52 (1.04–2.23)	
Dominant model		0.025*		0.026*
AA	1.00		1.00	
AC + CC	1.75 (1.07–2.85)		1.87 (1.08–3.24)	
Recessive model		0.789		0.77
AA + AC	1.00		1.00	
CC	1.10 (0.54–2.27)		1.13 (0.51–2.51)	

*: p-value < 0.05; a, After the adjustment for age and body mass index; OR, odds ratio; CI, confidence interval.

### mRNA expression of SNP polymorphisms with downstream genes

**Figure 2:**
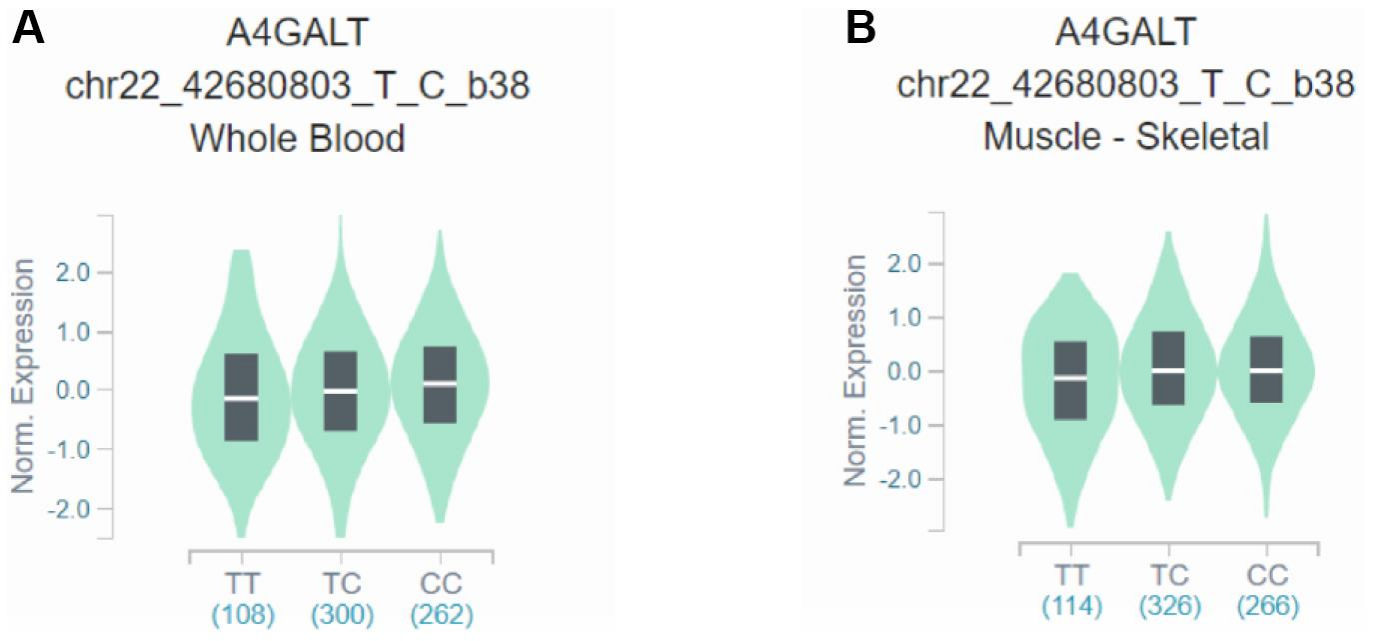
**Effects of rs130347 polymorphism on *A4GALT* expression.** (**A**) The presence of the rs130347 C minor allele in whole blood decreases downstream *A4GALT* expression (p = 0.0041) (**B**) In skeletal muscle tissue samples, the presence of the rs130347 C minor allele influences downstream *A4GALT* expression (p = 0.011).

**Figure 3:**
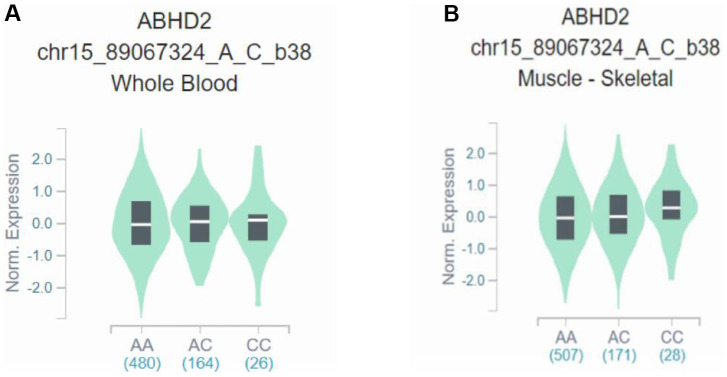
**Effects of rs28481460 polymorphism on *ABHD2* expression.** (**A**) The presence of the rs28481460 C minor allele in whole blood does not influence downstream *A4GALT* expression (p = 0.90). (**B**) The presence of the rs130347 C minor allele in skeletal muscle tissue samples does not influence downstream *ABHD2* expression (p = 0.68).

This case–control study showed that rs28481460 and rs130347 are associated with the risk of osteoporosis. In this study, the GTEx database was used for expression quantitative trait loci analysis of the mRNA expression of SNP loci and downstream genes. The GTEx query steps used for gene expression analysis are shown in Supplementary File 2. We observed that the presence of the rs130347 C minor allele in whole blood decreases downstream *A4GALT* expression (p = 0.0041). In skeletal muscle tissue samples, the presence of the rs130347 C minor allele influences downstream *A4GALT* expression (p = 0.011; Figure 2). Furthermore, we noted that the presence of the rs28481460 C minor allele in whole blood does not influence downstream *ABHD2* expression. In the skeletal muscle tissue samples, the presence of the rs130347 C minor allele does not influence downstream *ABHD2* expression (p = 0.68; Figure 3).

## DISCUSSION

In this study, we investigated the association of the binding sites of seven TFs (E2F4, EGR1, JUN, Sp1, TCF7L2, TP53, and CTNNB1) with osteoporosis. Our study results revealed that a binding site SNP of the TF, JUN, rs130347, was significantly associated with osteoporosis. In addition, we explored the mRNA expression of rs130347 in the GTEx database and noted that both the whole blood and the skeletal muscle samples showed that the presence of the C allele in rs130347 decreases *A4GALT* expression.

Osteoporosis is known to be caused by an imbalance between osteoblasts and osteoclasts [33]. Currently, it is known that the biological pathway that influences osteoblasts is the RANK-OPG-RANKL pathway [34]. The binding of RANK and RANKL stimulates NF-κB activation and increases MAPK, JNK, ERK, and p38 activities, and these signaling pathways influence osteoclast formation [35]. JUN and FOS are members of the activator protein 1 (AP-1) family. JNK protease influences osteoblast differentiation by enhancing its binding with Ap-1 family proteins [36]. To validate the effects of JUN on bone growth, a previous study transplanted long bones that induce JUN synthesis into mice with an impaired immune system [37]. The results revealed a significant increase in the bones of the transplanted mice. This showed that JUN is vital to the development of the skeletal system.

rs130347 is located upstream of *A4GALT*, has a length of 29 kb, and is located at 22q13.2. Its primary function is to catalyze the conversion of galactose to lactosylceramide to synthesize globotriaosylceramide (Gb3) [38]. A study demonstrated that Gb3 can bind to verotoxin produced by *Escherichia coli* to induce apoptosis [39]. Gb3 was also shown to be able to prevent human immunodeficiency virus infection [40]. Moreover, other studies showed that a genetic defect in α-galactosidase in patients with Fabry disease leads to the aberrant accumulation of Gb3 in endothelial cells, causing kidney, heart, and cerebrovascular lesions, and decreases BMD. This results in an increased risk of osteoporosis in patients with Fabry disease [41, 42]. However, the association between *A4GALT* and osteoporosis remains unknown and the biological mechanisms involved are yet to be elucidated. We found that rs130347 is a polymorphic locus located in the binding site of the TF, JUN, and causes a decrease in *A4GALT* expression.

In this study, we employed a candidate screening method different from previous studies and used the NGS data in the Taiwan BioBank to screen for TFBS polymorphisms that had the highest association with osteoporosis. SNP loci that were not previously investigated for their association with osteoporosis were identified in the case–control study. Compared with the results of GWAS, most SNPs found were not causally related and no association could be found with the disease [43, 44]. The bioinformatic analysis used in this study successfully identified one SNP that is correlated with osteoporosis. In future, multiple omics technologies, including genomics, transcriptomics, epigenomics, proteomics, and metabolomics, can be combined to identify the molecular factors contributing to disease pathogenesis and thus address genetic susceptibility to disease development.

Certain potential limitations of this study might have influenced the results. The differential gene data for candidate TFs were obtained from the mesenchymal stem cells of the Caucasian population, which may differ from the RNA sequencing results of the Asian population; this may have influenced the candidate TF results. Furthermore, this study used ChIP-Seq data from the JASPAR database for repeated validation of tissue-derived nonosteocytes, which may have influenced the results. Sample size limitations resulted in an inability to examine the effects of genes with an MAF of <5% and structural mutations (insertions/deletions) on osteoporosis. Therefore, we recommend increasing the sample size in the future to examine SNPs that were not covered in this study in order to obtain more osteoporosis-related SNP results.

## CONCLUSIONS

In summary, our data demonstrated that rs130347 plays an important role in postmenopausal women with susceptibility to osteoporosis, modulating the epigenetic regulation of a critical osteoporosis-related gene, *A4GALT*. rs130347 impairs the binding of JUN, which may lead to decreased *A4GALT* expression. However, the effect of the JUN binding site SNP rs130347 and *A4GALT* on the development and function of osteoporosis remains incompletely understood, and further exploration of the regulatory mechanism, such as by RNA-Seq of the genomes of Taiwanese patients, is warranted.

## Supplementary Material

Supplementary Figure 1

Supplementary File 1

Supplementary File 2
