# Supplementary figures and images for: Differential gene expression orchestrated by transcription factors in osteoporosis: bioinformatics analysis of associated polymorphism elaborating functional relationships
Source: Aging (Albany NY). 2022 Jun 21;14(12):5163–76. doi: 10.18632/aging.204136 (PMC9271311; doi:10.18632/aging.204136)

SUPPLEMENTARY FIGURE

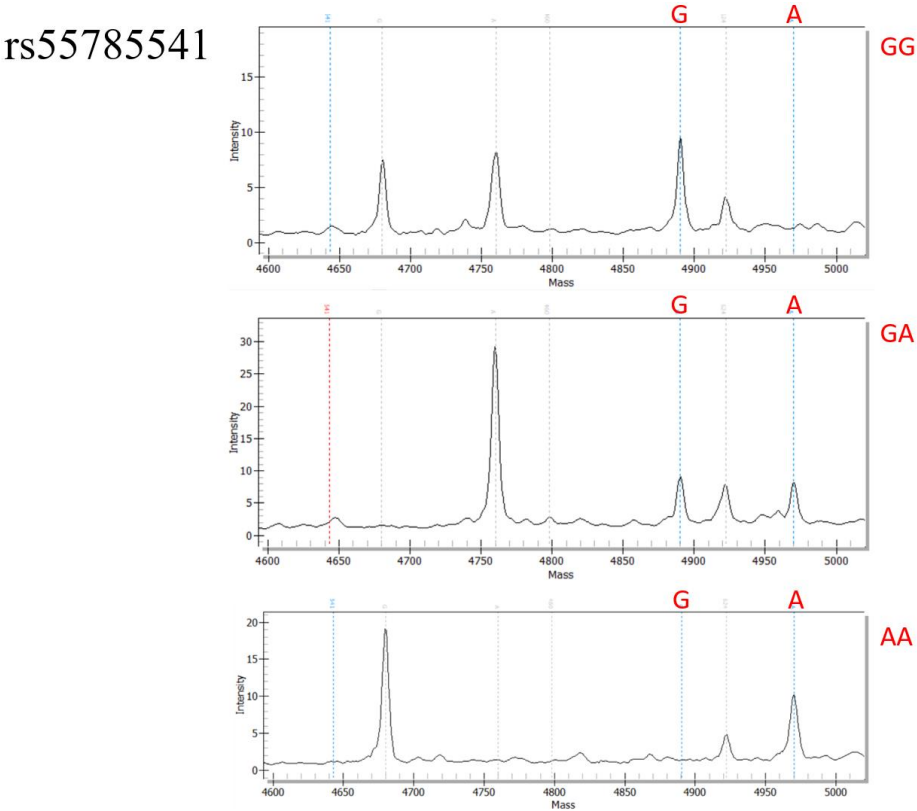

Supplementary Figure 1. Results of rs55785541 genotyping.

Supplement: Supplementary Figure 1 [file aging-14-204136-s001.pdf]
