# Supplementary material for: Differential gene expression orchestrated by transcription factors in osteoporosis: bioinformatics analysis of associated polymorphism elaborating functional relationships
Source: Aging (Albany NY). 2022 Jun 21;14(12):5163–76. doi: 10.18632/aging.204136 (PMC9271311; doi:10.18632/aging.204136)
Supplement: Supplementary File 1 [file aging-14-204136-s002.docx]

Supplementary File 1. Bioinformatics sequence alignment R code.

library(data.table)

library(magrittr)

library(dplyr)

library(plyr)

#5'-TGASTCA-3' (JUN)

NFkB.seq = "TGA[CG]TCA"

out.path = "data/JUN_AT30_CG20"

tfbs.path = "http://jaspar.genereg.net/download/bed_files/MA0488.1.bed"

length.NFkB.seq = 13

#0. Build gene ref

if (file.exists("data/Gene_Location.RData")) {load("data/Gene_Location.RData")} else {

library(BSgenome.Hsapiens.UCSC.hg19)

library(TxDb.Hsapiens.UCSC.hg19.knownGene)

HGNC.URL = "http://www.genenames.org/cgi-bin/download?col=gd_hgnc_id&col=gd_app_sym&col=gd_app_name&col=gd_prev_sym&col=gd_aliases&col=gd_pub_eg_id&status=Approved&status_opt=2&where=&order_by=gd_app_sym_sort&format=text&limit=&hgnc_dbtag=on&submit=submit"

HGNC.ref = fread(HGNC.URL, data.table = FALSE)

HGNC.ref = HGNC.ref[,c(6, 2, 3)]

txdb <- TxDb.Hsapiens.UCSC.hg19.knownGene

gn <- sort(genes(txdb))

GN = data.table(gene.num = gn@ranges@NAMES,

chr = as.data.frame(gn)[,1],

start = gn@ranges@start,

end = gn@ranges@start + gn@ranges@width - 1,

width = gn@ranges@width)

GN = GN %>% filter(as.character(as.data.frame(gn)[,1]) %in% paste0("chr", c(1:22, "X", "Y"))) %>% setDT()

GN = GN %>% mutate(chr = mapvalues(chr, paste0("chr", c(1:22, "X", "Y")), 1:24)) %>% setDF()

GN[,1] = as.integer(GN[,1])

GN[,2] = as.integer(GN[,2])

GN[,4] = as.integer(GN[,4])

GN = merge(GN, HGNC.ref, by.x = "gene.num", by.y = "Entrez Gene ID", all.x = TRUE)

GN = GN[order(GN[,3]),]

GN = GN[order(GN[,2]),]

colnames(GN)[6:7] = c("Short.name", "Full.name")

URL = "https://www.ncbi.nlm.nih.gov/gene/"

pb = txtProgressBar(max = nrow(GN), style = 3)

for (i in 1:nrow(GN)) {

if (is.na(GN[i,6])) {

sep.URL = paste0(URL, GN[i,1])

txt = scan(sep.URL, what="character", encoding="UTF-8", quiet = TRUE)

txt = paste(txt, sep="", collapse=" ")

pos.1 = regexpr('Gene symbol </dt> <dd class=\"noline\">', txt)[1] + 37

subtxt.1 = substr(txt, pos.1, pos.1 + 200)

pos.2 = regexpr('</dd>', subtxt.1)[1] - 1

GN[i,6] = substr(subtxt.1, 1, pos.2)

pos.3 = regexpr('Gene description </dt> <dd>', txt)[1] + 27

subtxt.2 = substr(txt, pos.3, pos.3 + 200)

pos.4 = regexpr('</dd>', subtxt.2)[1] - 1

GN[i,7] = substr(subtxt.2, 1, pos.4)

}

setTxtProgressBar(pb, i)

}

close(pb)

pb = txtProgressBar(max = nrow(GN), style = 3)

for (i in 1:nrow(GN)) {

if (GN[i,6] == "") {

sep.URL = paste0(URL, GN[i,1])

txt = scan(sep.URL, what="character", encoding="UTF-8", quiet = TRUE)

txt = paste(txt, sep="", collapse=" ")

pos.1 = regexpr('Official Symbol</dt> <dd class=\"noline\">', txt)[1] + 40

subtxt.1 = substr(txt, pos.1, pos.1 + 200)

pos.2 = regexpr('<span', subtxt.1)[1] - 1

GN[i,6] = substr(subtxt.1, 1, pos.2)

pos.3 = regexpr('Official Full Name</dt> <dd>', txt)[1] + 28

subtxt.2 = substr(txt, pos.3, pos.3 + 200)

pos.4 = regexpr('<span', subtxt.2)[1] - 1

GN[i,7] = substr(subtxt.2, 1, pos.4)

}

setTxtProgressBar(pb, i)

}

close(pb)

save(GN, file = "data/Gene_Location.RData")

write.csv(GN, "data/Gene_Location.csv", quote = FALSE, na = "", row.names = FALSE)

}

#0. Build tfbs ref

if (file.exists(paste0(out.path, "/tfbs.RData"))) {load(paste0(out.path, "/tfbs.RData"))} else {

tfbs = fread(tfbs.path)

colnames(tfbs) = c("Chrom", "start", "end", "name", "V5", "strand")

tfbs = tfbs %>% mutate(Chrom = as.integer(factor(Chrom, levels = paste0("chr", c(1:22, "X", "Y"))))) %>% setDF()

tfbs$gene = NA

tfbs$gene.num = NA

tfbs$gene.start = NA

tfbs$gene.end = NA

tfbs$coding = NA

for (k in 1:nrow(tfbs)) {

subGN = GN[GN[,2] == tfbs[k,1],]

pos1 = which(diff((tfbs[k,2] - subGN[,3]) < 0) == 1)[1] + 1

pos2 = which(diff((tfbs[k,2] - subGN[,4]) < 0) == 1)[1] + 1

if (!is.na(pos2)) {

tfbs[k,11] = (subGN[pos2,3] < tfbs[k,2] & subGN[pos2,4] > tfbs[k,3])

if (tfbs[k,11]) {

POS = pos2

} else {

POS = pos1

}

tfbs[k,7] = subGN[POS,6]

tfbs[k,8] = subGN[POS,1]

tfbs[k,9] = subGN[POS,3]

tfbs[k,10] = subGN[POS,4]

} else {

tfbs[k,11] = FALSE

}

}

tfbs$POS = tfbs[,2] - tfbs[,9]

tfbs = tfbs[,-c(4:5)]

save(tfbs, file = paste0(out.path, "/tfbs.RData"))

}

###################################################################

NGS.list = list()

for (j in 24:1) {

t1 = Sys.time()

#1. Read data

load(paste0("data/TWBNGS-Seq_chr", j, ".RData"))

#2. Data processing

FINAL.DATA = FINAL.DATA %>% mutate(Seq.1 = gsub("[", "", Seq.1, fixed = TRUE),

Seq.2 = gsub("[", "", Seq.2, fixed = TRUE),

Seq.3 = gsub("[", "", Seq.3, fixed = TRUE),

Seq.4 = gsub("[", "", Seq.4, fixed = TRUE)) %>% setDT()

FINAL.DATA = FINAL.DATA %>% mutate(Seq.1 = gsub("]", "", Seq.1, fixed = TRUE),

Seq.2 = gsub("]", "", Seq.2, fixed = TRUE),

Seq.3 = gsub("]", "", Seq.3, fixed = TRUE),

Seq.4 = gsub("]", "", Seq.4, fixed = TRUE)) %>% setDT()

FINAL.DATA = FINAL.DATA %>% mutate(Seq.1 = gsub("-", "", Seq.1, fixed = TRUE),

Seq.2 = gsub("-", "", Seq.2, fixed = TRUE),

Seq.3 = gsub("-", "", Seq.3, fixed = TRUE),

Seq.4 = gsub("-", "", Seq.4, fixed = TRUE)) %>% setDT()

FINAL.DATA = FINAL.DATA %>% mutate(Seq.1 = gsub("<(A|T|C|G)*>", "", Seq.1),

Seq.2 = gsub("<(A|T|C|G)*>", "", Seq.2),

Seq.3 = gsub("<(A|T|C|G)*>", "", Seq.3),

Seq.4 = gsub("<(A|T|C|G)*>", "", Seq.4)) %>% setDT()

FINAL.DATA.SNP = FINAL.DATA %>% filter(VarType == "SNP") %>% setDT()

FINAL.DATA.INS = FINAL.DATA %>% filter(VarType == "INS") %>% setDT()

FINAL.DATA.DEL = FINAL.DATA %>% filter(VarType == "DEL") %>% setDT()

FINAL.DATA.DEL = FINAL.DATA.DEL %>% mutate(NCHAR.1 = nchar(Seq.1)-1,

NCHAR.2 = nchar(Seq.2)-1,

NCHAR.3 = nchar(Seq.3)-1,

NCHAR.4 = nchar(Seq.4)-1) %>% setDT()

FINAL.DATA.DEL = FINAL.DATA.DEL %>% mutate(Seq.1 = substr(Seq.1, 1, NCHAR.1),

Seq.2 = substr(Seq.2, 1, NCHAR.2),

Seq.3 = substr(Seq.3, 1, NCHAR.3),

Seq.4 = substr(Seq.4, 1, NCHAR.4)) %>% setDT()

FINAL.DATA.DEL = FINAL.DATA.DEL[, 1:18, with = FALSE]

NGS.DATA = bind_rows(FINAL.DATA.SNP, FINAL.DATA.INS, FINAL.DATA.DEL)

#2. Matching

NGS.DATA.Comparison = NGS.DATA %>% mutate(Seq1.bind = grepl(NFkB.seq, Seq.1),

Seq2.bind = grepl(NFkB.seq, Seq.2),

Seq3.bind = grepl(NFkB.seq, Seq.3),

Seq4.bind = grepl(NFkB.seq, Seq.4)) %>% setDT()

NGS.DATA.Comparison = NGS.DATA.Comparison %>% mutate(L1 = nchar(Seq.1), L2 = nchar(Seq.2), L3 = nchar(Seq.3), L4 = nchar(Seq.4)) %>% setDT()

NGS.DATA.Comparison = NGS.DATA.Comparison %>% mutate(Included.bind.500 = (Seq1.bind == "TRUE"|Seq2.bind == "TRUE"|Seq3.bind == "TRUE"|Seq4.bind == "TRUE")) %>% setDT()

NGS.DATA.Comparison = NGS.DATA.Comparison %>% mutate(Seq.1 = paste0(substr(Seq.1, 500 - length.NFkB.seq + 2, 500), substr(Seq.1, 501, L1 - 500), substr(Seq.1, L1 - 499, L1 - (500 - length.NFkB.seq + 1))),

Seq.2 = paste0(substr(Seq.2, 500 - length.NFkB.seq + 2, 500), substr(Seq.2, 501, L2 - 500), substr(Seq.2, L2 - 499, L2 - (500 - length.NFkB.seq + 1))),

Seq.3 = paste0(substr(Seq.3, 500 - length.NFkB.seq + 2, 500), substr(Seq.3, 501, L3 - 500), substr(Seq.3, L3 - 499, L3 - (500 - length.NFkB.seq + 1))),

Seq.4 = paste0(substr(Seq.4, 500 - length.NFkB.seq + 2, 500), substr(Seq.4, 501, L4 - 500), substr(Seq.4, L4 - 499, L4 - (500 - length.NFkB.seq + 1)))) %>% setDT()

NGS.DATA.Comparison = NGS.DATA.Comparison %>% mutate(Seq1.bind = grepl(NFkB.seq, Seq.1),

Seq2.bind = grepl(NFkB.seq, Seq.2),

Seq3.bind = grepl(NFkB.seq, Seq.3),

Seq4.bind = grepl(NFkB.seq, Seq.4)) %>% setDT()

NGS.DATA.Comparison = NGS.DATA.Comparison %>% mutate(L1 = nchar(Seq.1), L2 = nchar(Seq.2), L3 = nchar(Seq.3), L4 = nchar(Seq.4)) %>% setDT()

NGS.DATA.Comparison = NGS.DATA.Comparison %>% mutate(diff.bind = ((n.Ale == 2 & !(Seq1.bind == Seq2.bind))|(n.Ale == 3 & !(Seq1.bind == Seq2.bind & Seq2.bind == Seq3.bind))|(n.Ale == 4 & !(Seq1.bind == Seq2.bind & Seq2.bind == Seq3.bind & Seq3.bind == Seq4.bind)))) %>% setDT()

NGS.DATA.Comparison = NGS.DATA.Comparison %>% mutate(Seq.1 = paste0(substr(Seq.1, 1, length.NFkB.seq - 1), "[", substr(Seq.1, length.NFkB.seq, L1 - length.NFkB.seq + 1), "]", substr(Seq.1, L1 - length.NFkB.seq + 2, L1)),

Seq.2 = paste0(substr(Seq.2, 1, length.NFkB.seq - 1), "[", substr(Seq.2, length.NFkB.seq, L2 - length.NFkB.seq + 1), "]", substr(Seq.2, L2 - length.NFkB.seq + 2, L2)),

Seq.3 = paste0(substr(Seq.3, 1, length.NFkB.seq - 1), "[", substr(Seq.3, length.NFkB.seq, L3 - length.NFkB.seq + 1), "]", substr(Seq.3, L3 - length.NFkB.seq + 2, L3)),

Seq.4 = paste0(substr(Seq.4, 1, length.NFkB.seq - 1), "[", substr(Seq.4, length.NFkB.seq, L4 - length.NFkB.seq + 1), "]", substr(Seq.4, L4 - length.NFkB.seq + 2, L4))) %>% setDT()

NGS.DATA.Comparison = NGS.DATA.Comparison %>% mutate(Seq.1 = mapvalues(Seq.1, "[]", ""),

Seq.2 = mapvalues(Seq.2, "[]", ""),

Seq.3 = mapvalues(Seq.3, "[]", ""),

Seq.4 = mapvalues(Seq.4, "[]", "")) %>% setDT()

NGS.DATA.Comparison = NGS.DATA.Comparison %>% mutate(Seq1.bind = as.character(Seq1.bind),

Seq2.bind = as.character(Seq2.bind),

Seq3.bind = as.character(Seq3.bind),

Seq4.bind = as.character(Seq4.bind)) %>% setDT()

NGS.DATA.Comparison.2 = NGS.DATA.Comparison %>% filter(n.Ale == 2) %>% setDT()

NGS.DATA.Comparison.3 = NGS.DATA.Comparison %>% filter(n.Ale == 3) %>% setDT()

NGS.DATA.Comparison.4 = NGS.DATA.Comparison %>% filter(n.Ale == 4) %>% setDT()

NGS.DATA.Comparison.2 = NGS.DATA.Comparison.2 %>% mutate(MAF = as.numeric(Freq.1)*(Seq1.bind=="TRUE") +

as.numeric(Freq.2)*(Seq2.bind=="TRUE")) %>% setDT()

NGS.DATA.Comparison.3 = NGS.DATA.Comparison.3 %>% mutate(MAF = as.numeric(Freq.1)*(Seq1.bind=="TRUE") +

as.numeric(Freq.2)*(Seq2.bind=="TRUE") +

as.numeric(Freq.3)*(Seq3.bind=="TRUE")) %>% setDT()

NGS.DATA.Comparison.4 = NGS.DATA.Comparison.4 %>% mutate(MAF = as.numeric(Freq.1)*(Seq1.bind=="TRUE") +

as.numeric(Freq.2)*(Seq2.bind=="TRUE") +

as.numeric(Freq.3)*(Seq3.bind=="TRUE") +

as.numeric(Freq.4)*(Seq4.bind=="TRUE")) %>% setDT()

NGS.DATA.Comparison.2 = NGS.DATA.Comparison.2 %>% mutate(Seq3.bind = "", Seq4.bind = "") %>% setDT()

NGS.DATA.Comparison.3 = NGS.DATA.Comparison.3 %>% mutate(Seq4.bind = "") %>% setDT()

NGS.Comparison = bind_rows(NGS.DATA.Comparison.2, NGS.DATA.Comparison.3, NGS.DATA.Comparison.4) %>% setDT()

NGS.Comparison = NGS.Comparison %>% dplyr::select(-L1) %>% dplyr::select(-L2) %>% dplyr::select(-L3) %>% dplyr::select(-L4) %>% setDT()

#NGS.Comparison = NGS.Comparison %>% mutate(MAF = 0.5 - abs(0.5 - MAF)) %>% setDT()

#3. Annotation

subGN = GN[GN[,2] == j,]

Coding.regien = list()

Promoter.regien = list()

for (i in 1:nrow(subGN)) {

Coding.regien[[i]] = subGN[i,3]:subGN[i,4]

Promoter.regien[[i]] = (subGN[i,3] - 1000):(subGN[i,3] - 1)

}

Coding.regien = unlist(Coding.regien)

Promoter.regien = unlist(Promoter.regien)

subtfbs = tfbs[tfbs[,1] == j,]

if (nrow(subtfbs)==0) {

Big.regien = NULL

Cis.regien = NULL

Trans.regien =NULL

} else {

Big.regien = list()

Cis.regien = list()

Trans.regien = list()

for (i in 1:nrow(subtfbs)) {

Big.regien[[i]] = (subtfbs[i,2]-100):(subtfbs[i,3]+100)

if (!is.na(subtfbs[i,4])) {

if (subtfbs[i,4] == "+") {

Cis.regien[[i]] = subtfbs[i,2]:subtfbs[i,3]

} else {

Trans.regien[[i]] = subtfbs[i,2]:subtfbs[i,3]

}

}

}

Big.regien = unlist(Big.regien)

Cis.regien = unlist(Cis.regien)

Trans.regien = unlist(Trans.regien)

}

NGS = NGS.Comparison %>% mutate(Coding = Position %in% Coding.regien * 2 + Position %in% Promoter.regien) %>% setDT()

NGS = NGS %>% mutate(Chip = Position %in% Cis.regien * 3 + Position %in% Trans.regien * 2 + Position %in% Big.regien) %>% setDT()

save(NGS, file = paste0(out.path, "/chr", j, ".RData"))

NGS.list[[j]] = NGS

t2 = Sys.time()

cat(paste0("The ",j," Chrom was end, we cost ",formatC(as.numeric(t2-t1, units = "secs"), 2, format = "f")," secs in this loop\n"))

NGS.list[[j]] = NGS

}

NGS.Final = bind_rows(NGS.list)

save(NGS.Final, file = paste0(out.path, "/NGS_all.RData"))

rm(list = ls()[which(!ls()%in%"NGS.Final")])

###################################################################

#source("https://bioconductor.org/biocLite.R")

#biocLite("BSgenome.Hsapiens.UCSC.hg19")

library(data.table)

library(magrittr)

library(dplyr)

library(plyr)

out.path = "data/MYOG_sarcopenia"

load(paste0(out.path, "/NGS_all.RData"))

#0. Build gene ref

if (file.exists("data/Gene_Location.RData")) {load("data/Gene_Location.RData")} else {

library(BSgenome.Hsapiens.UCSC.hg19)

library(TxDb.Hsapiens.UCSC.hg19.knownGene)

HGNC.URL = "http://www.genenames.org/cgi-bin/download?col=gd_hgnc_id&col=gd_app_sym&col=gd_app_name&col=gd_prev_sym&col=gd_aliases&col=gd_pub_eg_id&status=Approved&status_opt=2&where=&order_by=gd_app_sym_sort&format=text&limit=&hgnc_dbtag=on&submit=submit"

HGNC.ref = fread(HGNC.URL, data.table = FALSE)

HGNC.ref = HGNC.ref[,c(6, 2, 3)]

txdb <- TxDb.Hsapiens.UCSC.hg19.knownGene

gn <- sort(genes(txdb))

GN = data.table(gene.num = gn@ranges@NAMES,

chr = as.data.frame(gn)[,1],

start = gn@ranges@start,

end = gn@ranges@start + gn@ranges@width - 1,

width = gn@ranges@width)

GN = GN %>% filter(as.character(as.data.frame(gn)[,1]) %in% paste0("chr", c(1:22, "X", "Y"))) %>% setDT()

GN = GN %>% mutate(chr = mapvalues(chr, paste0("chr", c(1:22, "X", "Y")), 1:24)) %>% setDF()

GN[,1] = as.integer(GN[,1])

GN[,2] = as.integer(GN[,2])

GN[,4] = as.integer(GN[,4])

GN = merge(GN, HGNC.ref, by.x = "gene.num", by.y = "Entrez Gene ID", all.x = TRUE)

GN = GN[order(GN[,3]),]

GN = GN[order(GN[,2]),]

colnames(GN)[6:7] = c("Short.name", "Full.name")

URL = "https://www.ncbi.nlm.nih.gov/gene/"

pb = txtProgressBar(max = nrow(GN), style = 3)

for (i in 1:nrow(GN)) {

if (is.na(GN[i,6])) {

sep.URL = paste0(URL, GN[i,1])

txt = scan(sep.URL, what="character", encoding="UTF-8", quiet = TRUE)

txt = paste(txt, sep="", collapse=" ")

pos.1 = regexpr('Gene symbol </dt> <dd class=\"noline\">', txt)[1] + 37

subtxt.1 = substr(txt, pos.1, pos.1 + 200)

pos.2 = regexpr('</dd>', subtxt.1)[1] - 1

GN[i,6] = substr(subtxt.1, 1, pos.2)

pos.3 = regexpr('Gene description </dt> <dd>', txt)[1] + 27

subtxt.2 = substr(txt, pos.3, pos.3 + 200)

pos.4 = regexpr('</dd>', subtxt.2)[1] - 1

GN[i,7] = substr(subtxt.2, 1, pos.4)

}

setTxtProgressBar(pb, i)

}

close(pb)

pb = txtProgressBar(max = nrow(GN), style = 3)

for (i in 1:nrow(GN)) {

if (GN[i,6] == "") {

sep.URL = paste0(URL, GN[i,1])

txt = scan(sep.URL, what="character", encoding="UTF-8", quiet = TRUE)

txt = paste(txt, sep="", collapse=" ")

pos.1 = regexpr('Official Symbol</dt> <dd class=\"noline\">', txt)[1] + 40

subtxt.1 = substr(txt, pos.1, pos.1 + 200)

pos.2 = regexpr('<span', subtxt.1)[1] - 1

GN[i,6] = substr(subtxt.1, 1, pos.2)

pos.3 = regexpr('Official Full Name</dt> <dd>', txt)[1] + 28

subtxt.2 = substr(txt, pos.3, pos.3 + 200)

pos.4 = regexpr('<span', subtxt.2)[1] - 1

GN[i,7] = substr(subtxt.2, 1, pos.4)

}

setTxtProgressBar(pb, i)

}

close(pb)

save(GN, file = "data/Gene_Location.RData")

write.csv(GN, "data/Gene_Location.csv", quote = FALSE, na = "", row.names = FALSE)

}

#1. combine gene information

#load(paste0(out.path, "/NGS_all.RData"))

NGS.500 = NGS.Final %>% filter(Included.bind.500 == TRUE & VarType == "SNP" & call > 0.9) %>% setDT()

NGS.diff = NGS.500 %>% filter(diff.bind == TRUE) %>% setDT()

NGS.5 = NGS.diff %>% filter(MAF <= 0.95 & MAF >= 0.05) %>% setDT()

NGS.Chip = NGS.5 %>% filter(Chip > 1) %>% setDT()

NGS.Chip$gene = NA

NGS.Chip$gene.num = NA

NGS.Chip$gene.start = NA

NGS.Chip$gene.end = NA

NGS.Chip$coding.reg = NA

for (k in 1:nrow(NGS.Chip)) {

subGN = GN[GN[,2] == NGS.Chip[k,1],]

pos1 = which(diff((NGS.Chip[k,2] - subGN[,3]) < 0) == 1)[1] + 1

pos2 = which(diff((NGS.Chip[k,2] - subGN[,4]) < 0) == 1)[1] + 1

if (!is.na(pos2)) {

NGS.Chip[k,32] = (subGN[pos2,3] < NGS.Chip[k,2] & subGN[pos2,4] > NGS.Chip[k,2])

if (NGS.Chip[k,32]) {

POS = pos2

} else {

POS = pos1

}

NGS.Chip[k,28] = subGN[POS,6]

NGS.Chip[k,29] = subGN[POS,1]

NGS.Chip[k,30] = subGN[POS,3]

NGS.Chip[k,31] = subGN[POS,4]

} else {

NGS.Chip[k,32] = FALSE

}

}

NGS.Chip$POS = NGS.Chip$Position - NGS.Chip$gene.start
