# Supplementary material for: Differential gene expression orchestrated by transcription factors in osteoporosis: bioinformatics analysis of associated polymorphism elaborating functional relationships
Source: Aging (Albany NY). 2022 Jun 21;14(12):5163–76. doi: 10.18632/aging.204136 (PMC9271311; doi:10.18632/aging.204136)

Supplementary File 2. GTEx database gene expression query steps.

1. Go to GTEx database website (<https://gtexportal.org/>).
2. QTLs & Browsers > eQTL Calculator


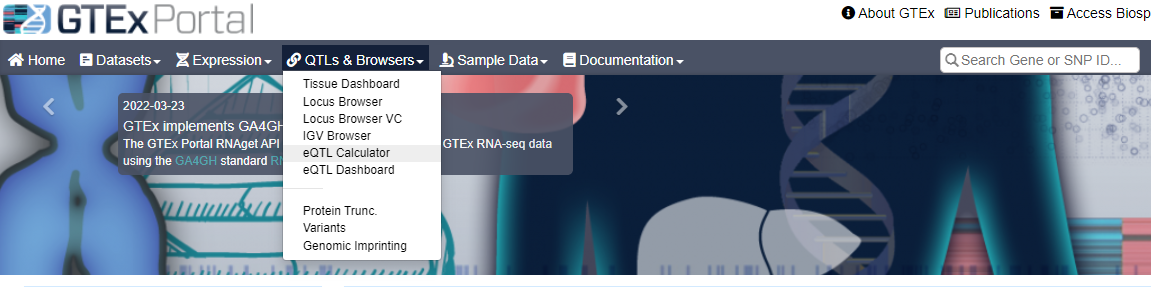


1. Enter Variant ID, Gene ID, Tissue name, then press “Calculate Your Own”.

Example:

rs28481460,ABHD2,Whole_Blood

rs28481460,ABHD2,Muscle_Skeletal


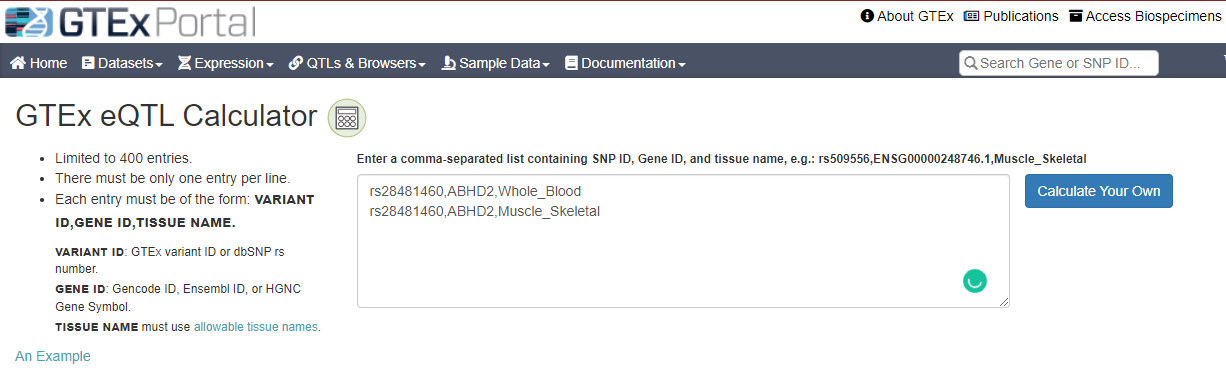


1. Obtain analysis results and eQTL violin plot.


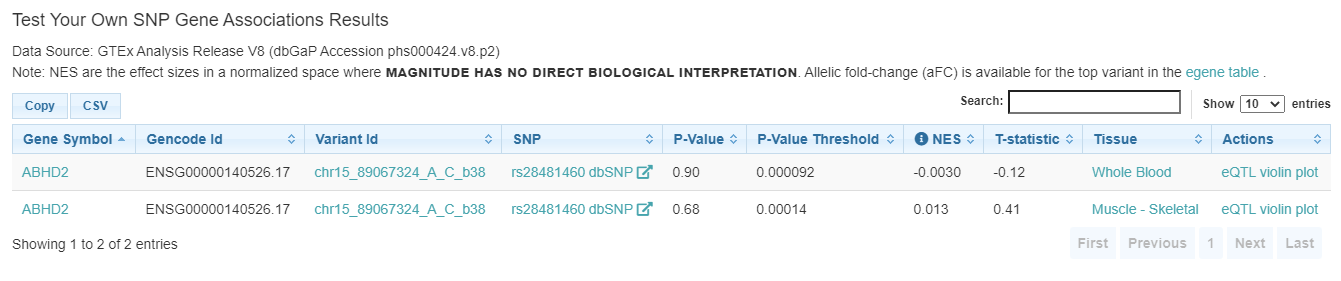

Supplement: Supplementary File 2 [file aging-14-204136-s003.docx]
